# Understanding the genetic diversity and population structure of yam (*Dioscorea alata* L.) using microsatellite markers

**DOI:** 10.1371/journal.pone.0174150

**Published:** 2017-03-29

**Authors:** Gemma Arnau, Ranjana Bhattacharjee, Sheela MN, Hana Chair, Roger Malapa, Vincent Lebot, Abraham K, Xavier Perrier, Dalila Petro, Laurent Penet, Claudie Pavis

**Affiliations:** 1 Unité Mixte de Recherche Amélioration Génétique et Adaptation des Plantes (UMR Agap), Centre de Coopération International en Recherche Agronomique pour le Développement (CIRAD), Station de Roujol, Petit Bourg, Guadeloupe, France; 2 Bioscience Center, International Institute of Tropical Agriculture (IITA), PMB, Ibadan, Oyo State, Nigeria; 3 Central Tuber Crops Research Institute (CTCRI), Sreekariyam, Triruvananthapuram, India; 4 Unité Mixte de Recherche Amélioration Génétique et Adaptation des Plantes (UMR Agap), CIRAD, Montpellier, France; 5 Vanuatu Agricultural Research and Technical Centre (VARTC), Espiritu Santo PB, Vanuatu; 6 Unité Mixte de Recherche Amélioration Génétique et Adaptation des Plantes (UMR Agap), CIRAD, Port-Vila, Vanuatu; 7 ASTRO Agrosystèmes Tropicaux, INRA, Petit-Bourg (Guadeloupe), France; National Cheng Kung University, TAIWAN

## Abstract

Yams (*Dioscorea* sp.) are staple food crops for millions of people in tropical and subtropical regions. *Dioscorea alata*, also known as greater yam, is one of the major cultivated species and most widely distributed throughout the tropics. Despite its economic and cultural importance, very little is known about its origin, diversity and genetics. As a consequence, breeding efforts for resistance to its main disease, anthracnose, have been fairly limited. The objective of this study was to contribute to the understanding of *D*. *alata* genetic diversity by genotyping 384 accessions from different geographical regions (South Pacific, Asia, Africa and the Caribbean), using 24 microsatellite markers. Diversity structuration was assessed via Principal Coordinate Analysis, UPGMA analysis and the Bayesian approach implemented in STRUCTURE. Our results revealed the existence of a wide genetic diversity and a significant structuring associated with geographic origin, ploidy levels and morpho-agronomic characteristics. Seventeen major groups of genetically close cultivars have been identified, including eleven groups of diploid cultivars, four groups of triploids and two groups of tetraploids. STRUCTURE revealed the existence of six populations in the diploid genetic pool and a few admixed cultivars. These results will be very useful for rationalizing *D*. *alata* genetic resources in breeding programs across different regions and for improving germplasm conservation methods.

## Introduction

Yams (*Dioscorea* sp.) are important food security crops for millions of small-scale farmers in the tropical and subtropical regions of Africa, Asia, the Pacific, the Caribbean and Latin America [1]. *Dioscorea alata* (known as the "greater yam" or the "winged yam") is one of the major cultivated species with wide geographical distribution [2]. It is currently second to *D*. *rotundata* in production volumes. Several traits of *D*. *alata* make it particularly valuable for commercial cultivation. These include high yield potential, ease of propagation, early growth vigour for weed suppression, and long storability of tubers [3, 4]. Tubers possess a high nutritional content with an average crude protein content of 7.4%, starch content of 75–84%, and vitamin C content ranging from 13.0 to 24.7 mg/100g [5].

*Dioscorea alata* is a dioecious species with a ploidy level ranging from 2n = 2x = 40 to 2n = 4x = 80 [6]. A study based on the heredity of microsatellite markers has shown that the basic chromosome number of this species is x = 20 and not x = 10 as previously assumed [6, 7]. This species was considered to be highly polyploid with six levels of ploidy (2n = 30, 40, 50, 60, 70 and 80) [8, 9]. However, it is now accepted that it has only three cytotypes (2n = 40, 60 and 80) and that the most common forms are diploids, followed by triploids and tetraploids are rare [6, 10, 11, 12].

Flowering of *D*. *alata* is erratic or absent in many cultivars [3, 13, 14, 15]. Cultivars have been exclusively clonally propagated by using small tubers or small pieces of tubers during hundreds or even thousands of years. Clonal propagation provides agronomical advantages but excludes sexual reproduction and could therefore represent a constraint for adaptation to biotic and abiotic stresses. It is also favorable to the spread of diseases, with pathogens allowed to adapt specifically to fixed genotype pools. The most serious disease in *D*. *alata* is anthracnose, which is caused by an airborne fungus *Colletotrichum gloesporioides* Penz. Anthracnose is found throughout the entire inter-tropical zone and can cause significant yield losses [16, 17, 18, 19, 20]. The importance of yams for food security has led to the establishment of several breeding programs for *D*. *alata*, in order to develop high-yielding cultivars with resistance to anthracnose, and tuber characteristics adapted to farmers’ requirements [2, 21, 22, 23]. Nevertheless, the lack of knowledge on its origin and genetic diversity limits the efficacy of genetic improvement.

The center of origin of *D*. *alata* is not known. Based on archaeological evidence, it is thought to have been domesticated ca. 6000 years ago and is native to Asia-Pacific, but is not known in its wild state [12]. The greatest phenotypic variability in *D*. *alata* was observed in the southern part of Southeast Asia and in Melanesia, the probable center of origin for this species [15, 24, 25, 26, 27]. The South Pacific islands (Papua New Guinea, Fiji, New Caledonia, the Solomon and Vanuatu islands) have rich *ex situ* collections of *D*. *alata*, including more than 1000 cultivars [12].

A wide diversity also exists in India [28, 29] where several *ex situ* germplasm collections were established, including the most important collection at CTCRI (Central Tuber Crops Research Institute, Kerala, India) with 431 accessions. In addition, several international collections have been assembled, including those of the CRB-PT (Centre de Ressources Biologiques Plantes Tropicales INRA-CIRAD, Guadeloupe, France) and the IITA (International Institute of Tropical Agriculture, Ibadan, Nigeria), with 181 and 772 accessions of *D*. *alata*, respectively.

Lebot et al. [27] evaluated the diversity within 269 accessions of *D*. *alata* from different regions (South Pacific, Asia, Africa and the Caribbean) using enzymatic markers. However, the weak polymorphism of the four enzymatic systems did not reveal correlations between the groups of zymotypes and the geographic origins, ploidy levels and/or the phenotypic characteristics of the accessions.

Various molecular markers have been used to characterize the genetic diversity of the *D*. *alata* collections, including RAPDs [30], AFLPs [31] and SSRs [32, 33, 34]. SSR markers (microsatellites) are considered to be the markers of choice for analyzing genetic diversity because of their co-dominance, high reproducibility, high global mutation rates and polymorphism [35, 36]. Nevertheless, the studies on *D*. *alata* involved a limited number of cultivars, and none was conducted at the global scale.

The aim of the present study was to analyze the genetic diversity and population structure of 384 *Dioscorea alata* accessions from different regions, including the South Pacific, Asia, Africa and the Caribbean using a common set of 24 microsatellite markers.

## Material and methods

### Plant material

Overall, 384 *D*. *alata* accessions originating from four collections were evaluated (S1 Table). These include two sets of germplasm: 363 landraces and 21 breeding lines, including 129 accessions from CRB-PT (Centre de Resources Biologiques Plantes Tropicales INRA-CIRAD, Guadeloupe), 90 from IITA (International Institute for Tropical Agriculture, Nigeria), 83 from CIRAD (Centre Internationale de Recherche Agronomique pour le developpement, Guadeloupe) and 82 from CTCRI (Central Tuber Crops Research Institute, India). The CIRAD collection is mainly composed of genotypes originating from Vanuatu (South Pacific). The accessions from IITA represented the core collection [37] developed from an entire collection of 772 *D*. *alata* West African landraces. The CRB-PT collection holds landraces from diverse geographical origins (Caribbean, South Pacific, South America). Overall, the germplasm was composed of 298 diploids, 51 triploids and 35 tetraploids. Ploidy levels of most accessions were determined in previous studies [6, 11, 31] except of 120 accessions (80 from IITA and 40 from CRB-PT), which were determined in this study using the protocol described in Arnau et al. [6].

### Genotyping

Genomic DNA of the accessions was isolated in each institute using the DNeasy Plant Mini Kit (Qiagen, Hilden, Germany) or the modified CTAB method as described by Sharma et al. [38].

Twenty-four SSR primer pairs (Table 1) developed from *D*. *abyssinica*, *D*. *praehensilis*, *D*. *japonica* and *D*. *alata*, were selected to analyze the accessions. Eleven primers were chosen based on their capacity to reveal high polymorphism and easy-to-score profiles (not stuttering) from a previous study (unpublished data, Arnau, 2013). The remaining thirteen were newly identified SSRs from *D*. *alata* [39]. Microsatellite alleles were scored using the software GeneMapper 4.0 (Applied Byosystems, USA).

**Table 1:** Genetic diversity detected in 367 D. alata accessions using 24 microsatellite markers. Ho, He and F*is* values were quantified only for diploids.

Origin ^1^	SSR	EMBL^2^	Motif	Min.–max. size (bp)	Total alleles	Al < 1%^3^	Main allele frequency^4^	Ho^5^	He^6^	Fis^7^
*D*. *A*	Da3G04	AJ880369	(AC)12	282–306	9	1	0.80	0.83	0.86	0.03
*D*. *A*	Da1F08	AJ880368	(TG)13	161–185	9	3	0.88	0.32	0.39	0.19
*D*. *A*	Da2F10	[51]	(TG)14	108–151	14	2	0.46	0.65	0.67	0.04
*D*. *A*	Da1A01	AJ880381	(GT)8	201–222	7	1	0.85	0.52	0.51	-0.02
*D*. *AB*	Dab2D11	[51]	(TC)19	227–247	9	2	0.66	0.83	0.67	-0.25*
*D*. *PR*	Dpr3E10	[51]	(TCT)13(CTC)4	170–194	10	1	0.84	0.16	0.32	0.16*
*D*. *PR*	Dpr3B12	AJ880376	(TG)8	132–150	8	2	0.81	0.74	0.65	-0.15
*D*.*J*	DIJ034	AB201419	(AG)17	198–267	15	2	0.48	0.90	0.80	-0.14
*D*.*J*	DIJ443	AB201420	(AG)17	257–285	12	0	0.45	0.73	0.82	0.11*
*D*.*J*	DIJ0461	AB201423	(GA)16	120–144	8	3	0.64	0.78	0.71	-0.10
*D*.*J*	DIJ1045	AB201422	(TG)19	251–281	13	4	0.52	0.78	0.71	-0.10
*D*.*A*	mDaCIR2	FN677762	(CA)10	243–268	9	0	0.44	0.78	0.71	-0.10
*D*.*A*	mDaCIR11	FN677767	(AC)10	165–183	8	4	0.79	0.47	0.54	0.12*
*D*.*A*	mDaCIR13	FN677768	(GA)17	186–214	14	4	0.49	0.56	0.79	0.28*
*D*.*A*	mDaCIR17	FN677770	(AC)7	228–236	5	2	0.96	0.18	0.20	0.11*
*D*.*A*	mDaCIR20	FN677773	(GA)16	172–206	11	1	0.69	0.67	0.62	-0.08
*D*.*A*	mDaCIR26	FN677776	(TG)15(GA)14	171–219	12	2	0.59	0.60	0.74	0.18
*D*.*A*	mDaCIR57	FN677784	(TG)9	143–152	5	0	0.86	0.83	0.55	-0.28*
*D*.*A*	mDaCIR59	FN677786	(TC)11(CA)9	186–224	12	5	0.55	0.75	0.75	-0.01
*D*.*A*	mDaCIR60	FN677787	(CA)11	132–159	12	1	0.56	0.62	0.81	0.23
*D*.*A*	mDaCIR25	[38]	(AC)14	142–184	14	1	0.39	0.49	0.81	0.34*
*D*.*A*	mDaCIR61	FN677788	(AG)21	179–221	18	5	0.61	0.81	0.74	-0.09
*D*.*A*	mDaCIR63	[38]	(AG)12	155–180	8	1	0.68	0.53	0.65	0.18*
*D*.*A*	mDaCIR116	FN677800	(AG)8(AG)7	83–126	14	3	0.70	0.61	0.65	0.06*

^1^D. A, D. alata; D. AB, D. abyssinica; D. PR, D. praehensilis; D. J, D. japonica

^2^ Registration number on EMBL database or publication reference

^3^ Rare alleles with a frequency lower than 1%

^4^ Highest frequency of an allele observed at this locus

^5^ Observed heterozygosity.

^6^ Expected heterozygosity

^7^ Fixation index,*P <0.001

PCR amplifications and gel electrophoresis were carried out on the GENTYANE genotyping platform (INRA UBP, UMR 1095, Clermont*-*Ferrand*)*. Each locus was fluorescently labeled by M13 tail as described by Vallunen [40]. Four different fluorochroms were used (FAM^TM^, VIC^R,^ NED^TM^ and PET^R^). The PCR amplifications were performed in a 10 μL final volume containing final concentrations of 1X AmpliTaq Gold 360 Master Mix (AB-life technologies), 0.05 μM labeled forward primer, 0.5 μM reverse primer and 25 ng of template DNA. For all loci the same PCR program was used, consisting of an initial denaturation at 95°C for 10 min followed by a touchdown PCR consisting of 45 cycles with denaturation at 95°C for 30 s; annealing for 30 s with temperature decreasing 1°C every cycle from 62°C to 56°C (7 cycles), then 30 cycles at 55°C and 8 cycles at 56°C; and a final extension at 72°C for 5 min. Two to four individual PCR products labeled with different fluorochroms were multiplexed and visualized using capillary gel electrophoresis on an ABI PRISM 3100 DNA sequencer (Applied Biosystems, Foster City, CA, USA).

### Data analysis

The genotypic data was converted into a binary matrix that recorded the presence (1) or absence (0) of alleles for each microsatellite locus per accession. The following genetic parameters were estimated: number of alleles per locus, number of rare alleles with a frequency lower than 1% [41], highest frequency of an allele observed for each locus, gene diversity (expected heterozygosity, He), observed heterozygosity (Ho) and fixation index (Fis). The latest three parameters were quantified only for diploid accessions using the Genepop 4.0.10 software [42]. To test for deviation from Hardy-Weinberg proportion, the exact test was used (500 batches and 5000 iterations). These parameters were not calculated for polyploid cultivars because of the difficulty of unambiguously determining allele dosage in polyploids [43].

In order to compare accessions and their relationships, we calculated genetic distances between each pair of accessions with Dice dissimilarity coefficients [44], using the NTSYSpc software version 2.1 [45]. We then proceeded with two approaches: first with a Principal coordinate analysis (PCoA) with the full study sample, and second a cluster analysis with UPGMA with a reduced sub-sample comprising all the diploid cultivars.

Indeed, since our study sample includes cultivars with three different ploidy levels (from diploids to tetraploids) whose parental contributions are unknown, we performed a Principal coordinate analysis (PCoA) to explore the diversity structure of all *D*.*alata* accessions. We used the Darwin software [46]. This method is indeed better than UPGMA to compare genetic diversity when plants with different ploidy levels are sampled [47].

We also carried out a cluster analysis for diploid accessions only using the UPGMA algorithm and the Dice similarity coefficient, using the NTSYSpc software version 2.1 [45]. Reliability and robustness of the clustering were based on 1000 random re-sampling conducted through the bootstrap procedure of TREECON software version 1.3. We also conducted a Mantel test [48] to assess the correlation between genetic distance and geographic origin of varieties for Vanuatu and Indian study varieties.

Population structure of diploids was also examined using a Bayesian approach using the software STRUCTURE V.2.3 [49]. This model assumes that the genome of individuals is a mixture of genes originating from K unknown ancestral populations. Under this model, the STRUCTURE algorithm estimated for each accession, the proportion of its genome (*q)* derived from the different clusters. So, individuals which may have mixed ancestry could be identified. In order to identify the number of populations (K) capturing the major structure in the data, we used the admixture model with a burn-in period of 30,000 steps and 10^6^ MCMC (Markov Chain Monte Carlo) replicates. The number of clusters (K) evaluated ranged from 1 to 10. The analysis was performed using three independent runs for each simulated value of K. We then calculated ΔK [50], an ad-hoc statistics based on the rate of change in the log probability of data between successive K values, to have an estimation of the real number of clusters. A threshold of q = 0.80 was used to assign genotypes to one of inferred K clusters. The molecular variance among populations and accessions within the populations were calculated using an Analysis of Molecular Variance (AMOVA) approach using the software ARLEQUIN V.3.5 [51].

## Results

Seventeen accessions presenting more than 25% of the missing data were removed from the analyses. The total number of accessions analyzed was 367, including 77 from India, 67 from Vanuatu, 89 from Caribbean, 87 from Africa, 13 from New Caledonia and 7 from French Guyana.

### Genetic diversity

The analysis of 24 SSRs across 367 accessions allowed the identification of 256 alleles (Table 1), with the number of alleles per locus ranging from five (loci mDaCIR17 and mDaCIR57) to 18 (locus mDaCIR61) with an average of 10.7 alleles per locus. The number of rare alleles present in less than 1% of the sample varied from zero to five per marker. The frequency of the most common allele at each locus varied from 0.39 to 0.96 (Table 1).

Genetic diversity (expected heterozygosity) values ranged from 0.20 to 0.86 and the observed heterozygosity from 0.16 to 0.90. The mean genetic diversity of 0.66 indicated moderate to high levels of polymorphism in *D*. *alata*. Nine loci showed a significant heterozygote deficit while two loci presented significant excess. Overall, a slight heterozygote deficit was observed across all accessions (F*is* = 0.03, P<0.001).

The Dice dissimilarity coefficients between all possible pairs of genotypes ranged from 0 and 0.86. Individuals with a distance of zero had the same alleles over the set of 24 loci and may be considered as possible duplicates. Several duplicates were detected (Table 2) in each of the collections. A set of six cultivars from three different germplasm collections [I-59, I-71, I-452 (CRB-PT), VU567, VU579 (CIRAD) and Da322 (CTCRI)] also showed identical genotypic profiles at 24 loci (Table 2).

**Table 2:** Details on duplicates from each collection based on genotypic profile across 24 SSR markers. The accessions grouped together presented identical allelic profiles at 24 SS loci.

Collection	Accession	Geographic	Local	Study
	code	origin	name	code
CTCRI	Da322	^4^India	Unknown	212
CIRAD	VU579	^3^Vanuatu	Letslets Bokis	318
CIRAD	VU567	^3^Vanuatu	Letslets Bolos	323
CRB-PT	PT-IG-00040	^2^Puerto Rico	59_Vino white forme	151
CRB-PT	PT-IG-00052	^2^Puerto Rico	71_Smooth Statia	168
CRB-PT	PT-IG-00395	Unknown	452_Fafadro bis	173
IITA	TDa-1427	^1^Ghana	Alamun Gaga	54
IITA	TDa-1437	^1^Ghana	Adidianmawoba	66
CTCRI	Da40	^4^India	Elivalan	192
CTCRI	Da73	^4^India	Muramchari	223
CTCRI	Da28	^4^India	Kachil	194
CTCRI	Da39	^4^India	Poolakachil	253
CTCRI	Da143	^4^India	Gutu	233
CTCRI	Da78	^4^India	Kachil	247
CTCRI	Da95	^4^India	Kudakachil	234
CTCRI	Da22	^4^India	Chuvanna Maveran	199
CTCRI	Da100	^4^India	Parisakodan	220
CTCRI	Da70	^4^India	Thekkan Kachil	222
CTCRI	Da120	^4^India	Kaduvakkayyan	228
CTCRI	Da105	^4^India	Chenithakizhangu	261
CTCRI	Da48	^4^India	Vila Kachil	255
CTCRI	Da209	^4^India	Kachil	243
CRB-PT	PT-IG-00061	^2^Martinique	80_Igname d eau	183
CRB-PT	PT-IG-00030	^2^Martinique	48_67	186
CRB-PT	PT-IG-00045	^2^Martinique	64_St Vincent Violet	370
CRB-PT	PT-IG-00067	^3^New Caledonia	86_Wénéféla bis	374

^1^Africa

^2^Caribbean

^3^South Pacific

^4^Asia

### Genetic structure

**Fig 1A:**
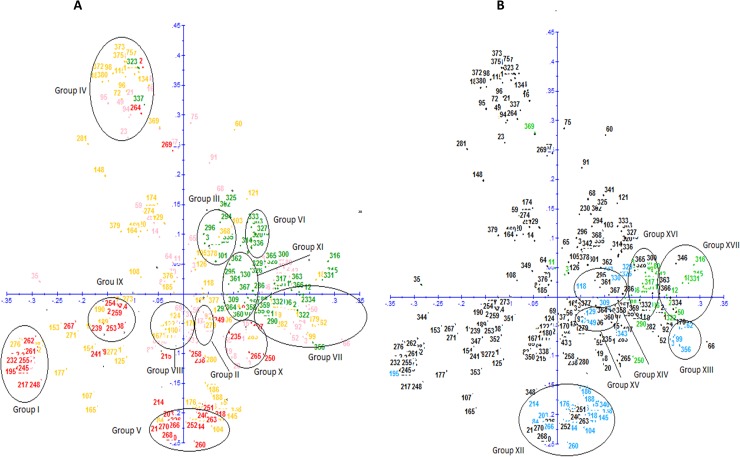
Diagram showing the relationships among 367 accessions of *D*. *alata* based on Principal Coordinate Analysis (PCoA) using 24 microsatellites. Clones originated from Vanuatu are colored in green and those from CTCRI (India) in red. The accessions of the CRB-PT and the IITA are colored in orange and pink, respectively. **1B. Diagram showing the relationships among 83 polyploid accessions of *D*. *alata* based on Principal Coordinate Analysis(PCoA) using 24 microsatellites.** Tetraploids accessions are colored in green and triploids in blue.

The population structure was first inferred by Principal coordinate analysis (PCoA) on all 376 accessions based on 256 alleles. The first two axes explained 57% of the genetic variability (Fig 1A and 1B).

Results showed that the accessions originating from India are primarily distributed in the lower part of the graph, whereas the accessions from Vanuatu are mainly distributed on the upper right-hand side (Fig 1A) revealing two distinct genepools. The Dice distances within the Indian collection ranged from 0 to 0.77, with an average value of 0.47 while that between Vanuatu cultivars varied from 0 to 0.79, with an average of 0.49. The genetic distances between the Indian cultivars and those from Vanuatu ranged from 0.31 to 0.82, with an average value of 0.61. This value is significantly higher than the average value within each of the collections. Moreover, some of the alleles are specific to each of these two genepools. Twenty-nine alleles present in the Indian collection are absent from the Vanuatu genepool, including 12 rare alleles. Similarly, fifty-five alleles present in the Vanuatu genepool are absent in the Indian collection, including 15 rare alleles. Our Mantel test for these two pools demonstrated that correlation between genetics and origin was very high (r = 0.408, *P* < 0.0001).

The accessions of the international collections (IITA and CRB-PT) are distributed almost everywhere on the PCoA, showing that the diversity observed in India and in Vanuatu is well represented in CRB-PT and partially represented in IITA (Fig 1A). PCoA also showed that cultivars originating from New Caledonia (South Pacific) from CRP-PT collection are mostly distributed on the same part of the graph (side upper right-hand) as the Vanuatu’s cultivars (South Pacific).

The 83 polyploid cultivars from the four collections (49 triploids and 34 tetraploids, S1 Table) are distributed in different zones of the PCoA graph and within proximity of the diploid cultivars (Fig 1B).

The combined analysis of PCoA and Dice distances allowed the identification of a total of seventeen different groups of cultivars composed of closely related cultivars (Fig 1A, Fig 1B and S1 Table) including eleven groups of diploids, four groups of triploids and two groups of tetraploids. The genetic distances between cultivars within each of these groups are lower than 0.25.

**Fig 2:**
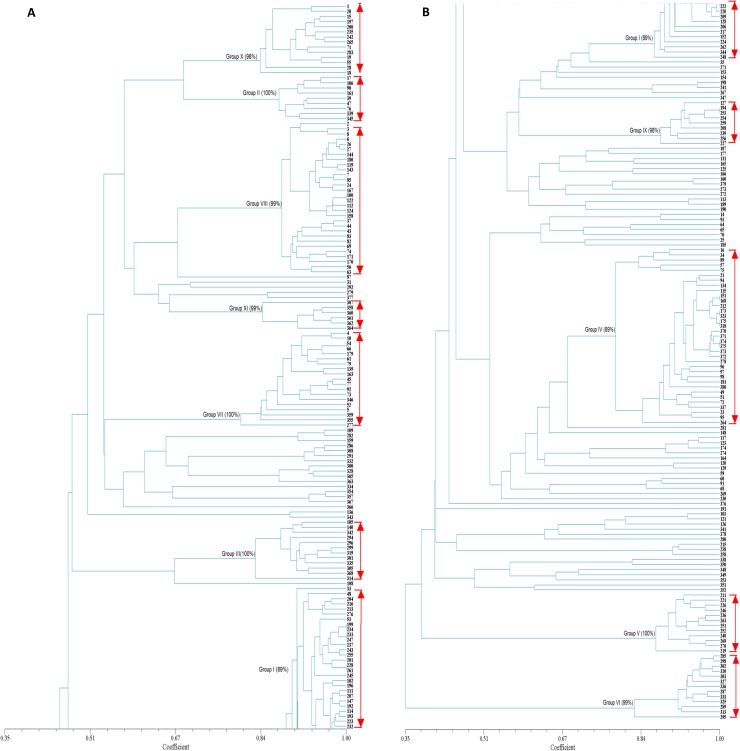
Dendrogram showing the relationships among 284 diploid accessions of *D*. *alata* based on UPGMA analysis using 24 microsatellites.

The UPGMA analysis supported the results of PCoA and clustered the diploids into the same eleven groups (Fig 2). Each group was supported with high bootstrap values (≥ 89%), indicating high stability of relationships among the accessions. These groups thus reflected strong phylogenetic relatedness and can be seen as natural groupings based on the observed bootstrap values.

### Diploid groups

Groups I, IV and VIII contained the highest number of cultivars (Fig 1A and Fig 2). Group I assembled 39 cultivars including 29 from India, eight from Caribbean and two from Africa. This group included the majority of duplicates identified within the CTCRI collection (Da40 and Da73, Da22 and Da95, Da70 and Da100, Da105 and Da120, Da78 and Da143, Da48 and Da209). Male cultivars cultivated in the Caribbean under the names of Pyramide, Brazo Fuerte and Brésil are also in this group. Group IV assembled 35 varieties from all geographic regions analysed (17 are from Caribbean, eleven from Africa, three from Vanuatu, two from India, one from New Caledonia, and one from French Guyana). This group included six accessions from three different collections that presented identical allelic profiles across 24 loci (I-59, I-71, I-452, VU567, VU579 and Da322), and can be considered as duplicates. It also listed two cultivars grown in the Caribbean with the names of Saint Vincent Blanc (I-54) and Saint Vincent Violet (I-64). The Dice distance between these two cultivars was 0.12 with 10 allelic differences at five loci. Group VIII represented 31 cultivars of which 20 are from Africa, 7 from Caribbean and 4 from French Guyana. This group included the cultivar, Pacala, which is cultivated and highly appreciated in the Caribbean but shows high susceptibility to anthracnose.

Group VII consisted of 19 cultivars of which 12 are from Africa, five from Caribbean and two from Vanuatu. The accessions from Africa (IITA cultivars TDa1427 and TDa1437) represented identical profiles across all 24 SSR loci (genetic distance equal to 0) and can be considered as duplicates. This group included the Florido variety selected by Martin and Rhodes [52] in Mayaguez, Puerto Rico, and distributed throughout the world. It also contains two cultivars that were classified in Vanuatu in the morphotypes group called Convar M3. This group is characterized by short oval tubers with white flesh, crumbly when cooked and highly appreciated in Vanuatu [15].

Group X assembled 15 cultivars including 9 from Africa, five from India and one from Caribbean. Group III included 12 cultivars, eight of which are from Vanuatu, one from New Caledonia and three from Caribbean. This group represents accessions known and cultivated in the Caribbean under the names of Kabusa and Lupias. The eight cultivars from Vanuatu were classified by Malapa [15] in the morphotype group Convar M1 since they present similarities of their aerial vegetative traits. These cultivars have elongate leaves with pointed foliar lobes, the base of their stem often has thorns, and their wings are not well developed on the stems. A wide variability exists for tuber shape, which may be long and cylindrical or compact and oval and the tuber flesh colour may be white or purplish. In addition, these cultivars produce male flowers and have a good tolerance to anthracnose in Vanuatu ([15]. Several of these cultivars were also evaluated in the Caribbean and presented good tolerance to anthracnose in Guadeloupe and in Martinique, including the accession (VU639a) known under the Vanuatu name of Malalagi [53].

Groups V and VI presented each 12 cultivars belonging to one collection. Group V is composed exclusively of cultivars from India and Group VI of cultivars from Vanuatu. Cultivars of group VI were classified in the morphotype convar M7 by Malapa [15]. They are characterized by hastate leaves whose colour are light green to yellow at maturity, by the presence of highly developed and undulated wings on the stem, and have short tubers that are often ridged. These cultivars produce male flowers in an erratic manner. A wide variability exists for tuber flesh colour (white, purple, reddish) that is highly appreciated in Vanuatu for its elastic consistency when cooked. It has been observed that these cultivars have a good tolerance to anthracnose in Vanuatu [15]. Several of these cultivars were also evaluated in the Caribbean and had a good tolerance to anthracnose disease in Guadeloupe and in Haiti [53, 54]. The two closest cultivars (VU434a and VU487a) have only one allelic difference across all 24 SSR loci and represented a genetic distance of 0.01. These cultivars differ for their tuber flesh color, which is white in VU434a and purplish white in VU487a.

Group II contained 9 cultivars five of which were from Africa and four from the Caribbean region. This group is composed of cultivars known and cultivated in the Caribbean under the names of Kinabayo and Oriental that produce female flowers. Cultivar Oriental was resistant to anthracnose in Guadeloupe [11] and was used as a source of resistance to produce resistant hybrids [22]. Within this group, the genetic distance between two accessions bearing the same name from two different collections (Kinabayo from CRB-PT and Kinabayo from CIRAD) (0.15) was greater than the genetic distance between two cultivars with different names of CRB- PT collection (cultivars Oriental and Kinabayo), whose genetic distance was 0.08.

Group IX also assembled nine accessions of which eight are from India and one from Caribbean. Group XI assembled six cultivars from Vanuatu that were classified in the morphotype group, Convars M4 [15]. They are characterized by a wide variability in tuber shape which may be long, compact or irregular. In addition, these cultivars produce male flowers and presented a good tolerance to anthracnose in Vanuatu [15].

The remaining 82 diploids (61 cultivars and the 21 breeding lines) were either not grouped or clustered in small groups containing two to four cultivars.

### Triploid groups

A total of four triploid groups were identified based on PCoA and genetic distances analysis (Fig 1B and S1 Table). The largest Group XII included 25 cultivars of which 18 are from Caribbean, six from India, two from French Guyana and 1 from New Caledonia. Two cultivars (I-80 and I-48) have identical profiles across 24 loci and can be considered as duplicates. This group includes three cultivars that share the name Tahiti (I-88: "French" Tahiti; I-87: "cultivated" Tahiti; and I-621: "snake" Tahiti). However, none of these cultivars are genetically identical across 24 SSR loci. They have nine to ten allelic differences (on four or five loci) and a genetic distance that varied between 0.10 and 0.11. Furthermore, cultivars belonging to this group have a characteristic allelic profile with three alleles at loci L31 (257 bp-269bp-275bp) and CIR59 (199bp-203bp-221bp), which is specific to this group and which made it possible to identify these cultivars.

Group XIII included six cultivars of which three are from New Caledonia, two from Africa and one from Vanuatu. This group includes accessions known in the Caribbean under the name of Goana. All cultivars belonging to this group have a characteristic allelic profile with three alleles at loci L31 (257 bp-261bp-265bp) and L30 (224bp-230bp-234bp), which is specific to this group. Group XV and XIV were the smallest and contained four and three accessions, respectively.

### Tetraploid groups

Two tetraploid groups were identified based on PCoA and genetic distances analysis (Fig 1B and S1 Table). The largest assembled 19 cultivars (Group XVI) of which 13 are from Africa, five from Vanuatu and one from New Caledonia. These accessions were classified by Malapa [15] in the morphotype group convar M17. They are characterized by wide, cordated leaves and rounded lobes, stems covered with thorns at their base, and developed and undulated wings. The tubers are long and either cylindrical or irregular in shape with purple flesh. They produce female flowers and showed tolerance to anthracnose in Vanuatu [15].

Group XVII included six cultivars of which one was from New Caledonia and five from Vanuatu. These cultivars were classified by Malapa [15] in the morphotype group convar M8. They produce male flowers and show tolerance to anthracnose in Vanuatu [15]. Several of these cultivars were also evaluated in the Caribbean and presented good tolerance to anthracnose disease in Guadeloupe and in Haiti [53, 54].

### Bayesian analysis of population structure

**Fig 3:**
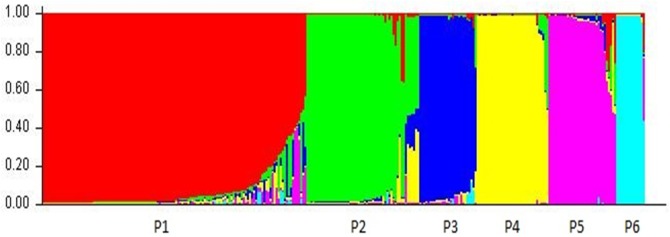
Structure of the genetic diversity of the 284 diploid accessions of *D*. *alata* at K = 6.

The Bayesian model approach implemented in STRUCTURE V2.3.4 allowed the analysis of the population structure of diploids (284 accessions). The Evanno’s method showed a peak of **Δk** for K = 6 supporting the presence of six genetically distinct populations (K = 6), here denoted as P1, P2, P3, P4, P5 and P6, respectively (Fig 3). Overall, 249 accessions (88%) were assigned to one of the six populations, where more than 80% of their inferred ancestry was derived from one of the model-based populations. P1 is composed of 115 accessions, among which 41 are from India, 50 from Caribbean, 20 from Africa and 4 from Vanuatu. This group is dominated by accessions originating from Indian genepool. P2 has 44 accessions, of which 28 are from Africa, seven from Caribbean, five from India and four from Vanuatu. This group corresponds to groups VII and X of PCoA. P3 included 20 accessions, among which 10 are from Caribbean and 10 from Vanuatu. P4 included 32 accessions, among which 20 are from Africa, 7 from Caribbean and 4 for French Guyana. These accessions corresponded to group VIII on PCoA. P5 included 25 accessions, among which three are from Caribbean, twelve from India, eight from Vanuatu and 1 from New Caledonia. These accessions corresponded to groups III and V on PCoA. P6 included 13 accessions and all originated from Vanuatu. These accessions are assigned to group VI on PCoA. The remaining 35 accessions (12%) included breeding lines and cultivars that showed admixed ancestry from different groups, including eight admixtures between P1 and P2, five between P2, P3 and P4, three between P1, P3 and P5, three between P2 and P4. The analysis of molecular variance (AMOVA) among populations indicated that 59.1% of the variation was due to differences within populations, while 40.9% was due to differences among populations (Table 3). The results of STRUCTURE analysis confirmed the existence of two divergent genepools in India and Vanuatu, and revealed some admixed cultivars, suggesting that these could have originated through hybridization between different populations. These results can be interpreted as population sets being clearly differentiated and with low historic admixture, to the exception of a few cultivars of recent origins. The groups defined by structure were thus possibly evolving and differentiating independently from each other with very low migration rates.

**Table 3:** Analysis of molecular variance (AMOVA) among populations and within populations.

Source of variation	df	SS	CV	%Total	P value
Among populations	5	1031.7	8.9	40.9	<0.0001
Within populations	138	1770.6	12.8	59.1	<0.0001
Total	143	2802.3	21.7	100	

df, Degrees of freedom; SS, Sum of squares; CV, Variance components estimation

% Total, percentage of total variation.

## Discussion

This study represents the first approach that comprehensively investigates the genetic diversity within different large collections of *D*. *alata* using microsatellite markers. Several other studies on genetic diversity have been conducted on this species using different types of molecular markers including random amplified polymorphism DNAs [30, 55], amplified fragment length polymorphisms [15, 56, 57] and simple sequence repeat (SSR) markers [34, 58]. However, these included accessions from only one or two regions or collections. Lebot et al. [27] evaluated the genetic diversity of 269 accessions of *D*. *alata* originating from the South Pacific, Asia and the Caribbean using isozymes. However, due to low polymorphism of these markers, no relationships could be established between these accessions.

Microsatellite markers were effective for identifying polymorphism and for evaluating the genetic relationships between the 367 accessions analyzed. Regarding all 24 SSRs loci investigated, the estimated diversity index (H’ = 0.66) was found to be high. Genetic diversity detected in this study is much higher than the one reported by Siqueira et al. [59] for 89 Brazilian *D*. *alata* cultivars (H’ = 0.41). The diversity level detected in this species is also higher than what is reported in 146 *D*. *rotundata* cultivars (H’ = 0.50) [60].

Our results revealed the existence of two divergent genepools in India and Vanuatu. In contrast to African species *D*. *rotundata*, whose center of origin is known and domestication is well documented [61, 62, 63], the center of origin for *D*. *alata* is actually not known yet. This species is supposed to have been domesticated in Southeast Asia [12] about 6000 years ago and then dispersed in India and the South Pacific Islands. Our results demonstrating a clear genetic differentiation between cultivars from India and Vanuatu are consistent with the existence of different secondary diversification centers in Asia and the South Pacific [12].

The introduction of *D*. *alata* in Africa and in New World takes place later in the 15^th^ and 16^th^ century. According to Prain and Burkill [64] this species was diffused from Asia to whole western Africa by the Portuguese in the 15^th^ century. Degras [13] argued that it was introduced into New World at the beginning of 16^th^ century with the slave trade.

PCoA and UPGMA analysis revealed a clear separation of the accessions into 17 major groups of genetically close cultivars, including eleven groups of diploids, four groups of triploids and two groups of tetraploids.

Two of the diploid groups assembled only cultivars from Vanuatu (VI and XI) and one group exclusively from India (V). The other diploid groups included cultivars from several geographic regions. Among these two included cultivars from India (I and X) and two from South Pacific (III and VII). One group (IV) assembled accessions from all geographic origins analyzed (Caribbean, Africa, Vanuatu, India, New Caledonia and French Guyana). It included six cultivars from three different geographic origins (Caribbean, India and Vanuatu) which are genetically identical across 24 loci. The results indicate that farmers adopt good yam cultivars with superior attributes readily and these eventually get widely distributed.

The four triploid groups included cultivars from several geographic regions. Three groups consisted of cultivars from Vanuatu and one had cultivars from India. The biggest group (XII) included cultivars that have been largely diffused (India, Caribbean, French Guyana and New Caledonia). One group (XIV) included only cultivars from South Pacific (Vanuatu and New Caledonia). Group XIV assembled cultivars from Africa and South Pacific (Vanuatu and New Caledonia), while group XV included cultivars from Caribbean and Vanuatu.

Both tetraploid groups contained cultivars from several geographic regions, except from India. Group XVI included cultivars from Africa and South Pacific (Vanuatu and New Caledonia) and Group XVII assembled cultivars only from South Pacific (Vanuatu and New Caledonia).

Our results demonstrated a high degree of differentiation within this highly heterozygous species, probably due to limited gene flow, primarily restricted by the mating system of this species. This work showed also that *D*. *alata* cultivars originating from Africa and from the New World (Caribbean and South America) are genetically very similar to Asian and/or South Pacific cultivars.

The reproductive system is one of the important life-history characteristics that strongly influence genetic variability [65]. *Dioscorea alata* cultivars are exclusively vegetatively-propagated and present a low fertility. Two different hypotheses could explain this structuration in several groups of genetically close cultivars:

1. Accessions belonging to the same group are the outcome of former sexual events with the same parents or genetically related parents.
2. Accessions belonging to the same group are the outcome of the same initial clone that evolved via somatic mutations fixed by vegetative propagation.

Given the mutational rate of SSR markers (between 10^−3^ and 10^−6^, depending on the species and on their position in the genome [36]) and the values of the genetic distances observed within these groups, it is likely that these two events contributed to the structuring of the genetic diversity in *D*. *alata*. The low number of allelic differences between cultivars assembled in group III, suggests the most likely hypothesis that this group was created from a single initial clone that evolved through mutations. Indeed, the number of allelic differences within this group corresponds to a total of 1 to 7 mutations, which is consistent with this hypothesis, on the basis of a mutation level of 10^−3^–10^−4^.

In contrast, the number of alleles differentiating the cultivars reassembled in other groups (ex: groups IV and VI) is higher to explain their origin from only an initial genotype. So, the most likely hypothesis is that these groups were created from several genetically related clones, which evolved through somatic mutations over time.

Our results are congruent with those obtained with DArTs to study the origin of *D*. *alata* cultivars in Vanuatu [66]. The low number of unique genotypes and the presence of numerous cultivars sharing a clonal origin generated a low varietal richness index (R = 0.26). This low index suggests that sexuality plays a minor role in the local diversification process. AFLPs have also highlighted the predominance of clonal reproduction in Vanuatu cultivars [15]. However, sexual recombination in *D*. *alata* appears to be rare in farmers’ fields and makes the diversification of local cultivars by selecting spontaneous seedlings difficult. However, farmers can detect somaclonal variants and propagate them. The high genetic variability observed between *D*. *alata* cultivars in Vanuatu is thought to be the results of multiple introductions of genetically distant individuals. The role of sexual reproduction and mutation in shaping the diversity has also been well documented for *D*. *rotundata* species [67].

Varietal diversity can be increased by controlled pollinations and breeding new cultivars and this has proven to be an effective way of genetic improvement [21, 22, 23, 68]. Most of the accessions included in our study are not fertile. Triploids are sterile [68, 69], as are several diploids and tetraploids. The results presented here allowed the identification of different groups of fertile cultivars with a good tolerance to anthracnose disease (Groups II, III, VI, XI, XVI and XVII) as well as several groups that contain cultivars known for the quality of their tubers: organoleptic, shape, dry matter content, and others (Groups III, VI, VII, VIII). These results will be useful for improving the germplasm management as well as for selecting genetically distant parents, even from different collections, to maximize allelic diversity and heterosis in breeding programs.

Much larger evaluations of *D*. *alata* genetic resources existing in Asian and South Pacific countries are still necessary in order to apprehend the genetic diversity available in these regions and allow their preservation and valorization in breeding programs.

## Conclusions

Microsatellite markers and a worldwide sample material proved to be effective for identifying polymorphism and for evaluating genetic relationships between yam varieties, clarifying relationships between genetic diversity, geographic origins, and ploidy levels.

We did not identify any center of origin for this crop, but we demonstrated in our study the existence of two diversification pools, one from Vanuatu, and the other from India. High diversity levels were found in international germplasm collections, with CRB collection spanning throughout worldwide genetic diversity, while IITA collection was spanning a lesser extent but targeted more specific diversity component of variation for this species, possibly reflecting a finer sampling scheme.

## Supporting information

S1 Table Details of accessions with their accession code, geographical origin, local name, ploidy level and accession type included in the study. Identified groups based on PcoA and UPGMA analysis are also indicated.
(XLSX)
